# Clinicobiochemical Parameters and Predictors of Liver Disease in Hospitalized Asian Indian Pregnant Women in a Tertiary Care Center in Northern India

**DOI:** 10.7759/cureus.13405

**Published:** 2021-02-17

**Authors:** Vishal Bhandari, Kamal Sharma, H S Pannu, Rajoo S Chhina, Ashima Taneja, Hardik D Desai, Neel N Patel, Khushboo N Patel, Sukriti Bhalla, Hardik Y Patel

**Affiliations:** 1 Cardiology, Tagore Hospital and Heart Care Center, Jalandhar, IND; 2 Cardiology, U N Mehta Institute of Cardiology and Research Center, Ahmedabad, IND; 3 Internal Medicine, Fortis Hospital, Ludhiana, IND; 4 Gastroenterology and Hepatology, Dayanand Medical College and Hospital, Ludhiana, IND; 5 Obstetrics and Gynaecology, Dayanand Medical College and Hospital, Ludhiana, IND; 6 Medicine, Gujarat Adani Institute of Medical Sciences, Bhuj, IND; 7 Medicine, B J Medical College, Ahmedabad, IND; 8 Medicine, C U Shah Medical College, Surendranagar, IND; 9 Cardiology, Akash Healthcare Super Specialty Hospital, New Delhi, IND

**Keywords:** liver disease, pregnant females, biochemical paramenter, clinical profile

## Abstract

Introduction

During pregnancy, liver dysfunction is more frequent than expected and may require specialized care. For the early diagnosis, it is important to determine if changes in liver physiology may develop into liver disease. Liver disease during pregnancy may require intervention from a hepatologist for adequate monitoring of mother-fetus health outcomes. This study was aimed to evaluate the clinical profile and predictors of maternal mortality in patients with liver diseases among Asian-Indian-females.

Methods

We conducted a prospective, open-label, consecutive all-comers study of 2,663 pregnant Asian Indian women admitted in the hospital, which included 92 with liver dysfunction. The medical aspects of the pregnancy were then followed prospectively with laboratory and clinical data during the hospital stay and analyzed. The current study was approved by the Institutional Ethical Committee.

Results

We found that 92 out of 2,663 patients had liver dysfunction with a prevalence of 3.45%. Fifty-four (58.7%) patients had icterus followed by fever in 23 (25.0%), hypertension in 22 (23.9%), central nervous system manifestations in 21 (22.8%), abdominal pain in 19 (20.6%), vomiting in 19 (20.6%), and pruritus in six (6.5%). Predictors of maternal mortality were icterus (p = 0.04), hepatomegaly (p = 0.04), presenting serum-bilirubin greater than 10 milligram% (mg%) (p = 0.008). The most common etiology was acute viral hepatitis (45.6%), followed by a hypertensive disorder of pregnancy (29.3%), acute fatty liver of pregnancy (1.1%), cholestatic jaundice (9.8%), hyperemesis gravidarum (2.2%), septicemic hepatitis (3.3%), dengue immunoglobulin M (IgM), and plasmodium vivax malaria antigen positive in (2.2%) each. Four patients (4.3%) were leptospira IgM reactive and had co-infection with hepatitis E virus. There was one patient (1.1%) with underlying chronic liver disease. Idiopathic liver disease was present in 5.4% of patients.

Conclusion

Liver disease is relatively common in Indian pregnant women. It is associated with high maternal and perinatal mortality, even in a tertiary referral center. When managing pregnancy in a tertiary care center, for adequate follow-up of the disease and to prevent adverse consequences for mother and child, it is important to discard liver alterations early. For this purpose, liver disease during pregnancy needs early diagnosis for proper management. Furthermore, it is difficult to manage patients with preexisting liver disease, and it may require specialized intervention from a hepatologist and a gastroenterologist.

## Introduction

Abnormal liver tests occur in 3-5% of pregnancies, with many potential causes, including coincidental liver disease (most commonly viral hepatitis or gallstones) and underlying chronic liver disease (CLD). In tropical countries like India, morbidity and mortality due to liver diseases in pregnancy are high [1,2]. In addition, morbidity is more likely in the presence of preexisting liver disease as in autoimmune hepatitis or when a new-onset liver disease occurs during pregnancy as in herpes simplex hepatitis [1].

Liver diseases in pregnancy can be classified as follows: (A) pregnancy-related liver diseases include (1) hyperemesis gravidarum, (2) intrahepatic cholestasis of pregnancy, (3) pre-eclampsia and eclampsia, (4) hemolysis, elevated liver enzymes, and low platelets (HELLP) syndrome, and (5) acute fatty liver of pregnancy (AFLP). (B) Pregnancy-unrelated liver diseases include (1) pre-existing liver diseases, (2) cirrhosis and portal hypertension, (3) hepatitis B and C, (4) autoimmune liver disease, (5) Wilson's disease, (6) liver diseases co-incident with pregnancy, (7) viral hepatitis, (8) biliary disease, (9) Budd-Chiari syndrome, (10) liver transplantation, (11) drug-induced hepatotoxicity, and (12) others including septicemic hepatitis, malaria, dengue, and leptospirosis [3]. While pregnancy-specific or pregnancy-predisposed hepatobiliary diseases are well described, their epidemiology in India is not well documented. We aimed to study the clinical profile and predictors of liver disease in Asian Indian pregnant women.

## Materials and methods

We conducted a prospective, open-label, consecutive all-comers study. Out of 2,663 screened Asian Indian pregnant women, 92 women with liver disease admitted to the hospital with liver dysfunction from January 2018 to June 2019 were included in the study. 

Study population

Inclusion criteria were (1) serum bilirubin >1.2 milligrams per deciliter (mg/dL) or (2) aspartate transaminase (AST) >40 units/liter (U/L) or (3) alanine transaminase (ALT) >40 U/L, or (4) alkaline phosphatase (ALP) >240 U/L as per the International Federation of Clinical Chemistry for the diagnosis of liver dysfunction. Exclusion criteria for the study were that pregnant women with any one of the following were excluded from the study: (1) isolated hyperbilirubinemia due to other causes like hemolysis and congenital diseases including Gilbert's syndrome, Dubin Johnson syndrome, Crigler-Najjar syndrome, Rotor syndrome; (2) accidental injury to the liver, including blunt trauma.

Clinical and laboratory workup

Pregnant women with diagnosed liver disease or suspected liver disease due to the presence of one or more of the following were further evaluated clinically for presenting symptoms and signs with special focus on icterus, abdominal pain, nausea/vomiting, itching, preeclampsia, clinical evidence of infection, and any sign or symptom suggestive of liver disease. A detailed history of the patient was recorded, and a thorough physical examination was carried out. The medical aspects of the pregnancy were then followed prospectively during the hospital stay and were investigated as clinically appropriate.

The following laboratory tests were carried out depending on the clinical presentation of the patient: complete hemogram (hemoglobin, total and differential count, platelet count), liver function test (total bilirubin, direct bilirubin, AST, ALT, ALP, total protein, serum albumin, albumin-globulin ratio, coagulation profile, such as prothrombin time, international normalized ratio (INR), bleeding time, clotting time, fasting blood sugar, random blood sugar, viral markers hepatitis B surface antigen, anti-hepatitis C virus, anti-human immunodeficiency viruses 1 and 2, ultrasonography (USG) abdomen, USG for fetal well-being. The following special investigations were done as indicated in individual cases: hepatitis B core antibody, immunoglobulin M (IgM), anti-hepatitis A virus or hepatitis E virus (HEV), serum ceruloplasmin, serum lactate dehydrogenase, serum gamma-glutamyl transferase, renal function test (serum creatinine, blood urea, serum electrolytes, namely sodium, potassium, and chloride ), serum uric acid, arterial ammonia, and urinary ketones.

These investigations were done and repeated as per the clinical requirement. The maternal and perinatal outcomes during the hospital stay were observed and recorded.

Statistical analysis

A descriptive analysis was performed on the collected data. Continuous variables were summarized as mean (and/or range), while number (percentage) and range were described as categorical variables. Data collected have been analyzed by using analysis of variance and student's t-test using statistical package for the social sciences (SPSS) software version 22 (IBM Corp., Armonk, NY, USA), and a two-sided p-value of less than 0.05 has been taken as significant.

## Results

Out of 2,663 pregnant women who were included in the study period between January 2011 and June 2012, 92 pregnant women were diagnosed with liver-related disease. This gave a prevalence of 3.45% of liver dysfunction among our cohort of pregnant women admitted to our hospital in the above-mentioned time period. Among the 92 pregnant women with liver disease, the majority (90.2%) were in the age group between 20 and 30 years. Forty-three patients (46.7%) were in the age group between 20 and 25 years and 40 patients (43.5%) were in the age group between 26 and 30 years. The majority of pregnant women (84.8%) who were diagnosed to have the liver-related disease were in the third trimester of their pregnancy (Table 1).

**Table 1:** Age distribution of subjects according to gestational age (n = 92)

Age (years)	14-28 weeks		29-40 weeks		Postpartum		Total	
	No.	%	No.	%	No.	%	No.	%
20-25	5	50.0	37	47.4	1	25.0	43	46.7
26-30	3	30.0	34	43.6	3	75.0	40	43.5
31-35	2	20.0	4	5.1	0	0.0	6	6.5
36-40	0	0.0	2	2.6	0	0.0	2	2.2
>40	0	0	1	1.3	0	0	1	1.1
Total	10	10.9	78	84.8	4	4.3	92	100

In our study, we found the majority (58.7%) of the patients presented to our hospital with symptoms of jaundice (58.7), followed by fever (25.0%), increased blood pressure readings (23.9%), central nervous system manifestations (22.8%), pain in the abdomen (20.6%), vomiting (20.6%), and pruritus (6.5%) (Table 2).

**Table 2:** Symptoms of liver disease in pregnancy and its relationship with maternal mortality (n = 92)

Chief complaints	No. of patients (%)	Maternal mortality n (%)
Jaundice	54 (58.7)	6/54 (11.1)
Fever	23 (25.0)	4/23 (17.4)
Increased blood pressure reading	22 (23.9)	2/22 (9.1)
Pain in the abdomen	19 (20.6)	-
Vomiting	19 (20.6)	4/19 (21.0)
Pruritus	6 (6.5)	-
Central nervous system manifestations	21 (22.8%)	3/21 (14.3)
Headache	8 (8.7)	-
Altered sensorium	6 (6.5)	1/6 (16.66)
Seizures	5 (5.4)	2/5 (40%)
Blurring of vision	2 (2.2)	-

At the time of presentation, 83 patients (90.2%) were fully conscious, while nine patients (9.8%) were stuporous. Maternal mortality was observed to be higher in those in a stuporous state (22.2%) but it was not statistically significant (p = 0.09). All seven maternal deaths were observed in a group of patients with icterus (12.1%), which was statistically significant (p = 0.04). Twenty patients (30.4%) had pallor found on general physical examination. No statistically significant (p = 0.94) change in maternal mortality has been observed in those with or without pallor. Hepatomegaly was present in 12 patients (13.0%) out of 92 pregnant women with liver disease. Maternal mortality was observed to be statistically significant (p = 0.04) in a group of patients with hepatomegaly (25.0%) than in those with no hepatomegaly (5.0%). Splenomegaly was present in four patients (4.3%) out of 92 pregnant women with liver disease. Maternal mortality was observed to be statistically insignificant (p = 0.12) in a group of patients with splenomegaly (25.0%) than in those with no splenomegaly (6.8%). Out of 92 patients included in the study, 37 patients (40.2%) had thrombocytopenia, including platelet counts less than 1.5 lakh per microliter (µL). Maternal mortality in those with thrombocytopenia (8.1%) was not significant (p = 0.91) compared to those with platelet count within normal limits (7.3%). Among the 92 pregnant patients who were admitted with liver-related diseases, 69 patients (75.0%) had a total bilirubin level of more than 1.2 mg/dL. Maternal mortality was statistically significant (p = 0.008) in those with total bilirubin more than 10.0 mg/dL (23.1%) compared with total bilirubin less than 10.0 mg/dL. Among 91 patients, 51 patients (56.04 %) had AST levels >120 U/L. Out of 83 patients in whom INR was done, 34 patients (41.0%) had coagulopathy, 34 out of 83 (40.96%) had INR >1.3, and maternal mortality was not statistically significant. Although, it is found to be trending toward significance (p = 0.059) (Table 3).

**Table 3:** Association of clinical signs and laboratory characteristics with maternal mortality SGPT: serum glutamic pyruvic transaminase; SGOT: serum glutamic-oxaloacetic transaminase; AST: aspartate aminotransferase;, ALT: alanine transaminase; ALP: alkaline phosphatase;  INR: international normalized ratio; μL: microliter; U/L: unit per liter; mg/dL: milligrams per deciliter

Variables	No. (%) (N = 92)	Maternal mortality (%)	Significance
Clinical signs			
Consciousness level			
Conscious	83 (90.2)	5/83 (6)	0.09
Drowsy/stupors/comatose	9 (9.8)	2/9 (22.2)	
Icterus	58 (63)	7/58 (12.1)	0.04
Pallor	28 (30.4)	2/28 (7.1)	0.94
Hepatomegaly	12 (13)	3/12 (25)	0.04
Splenomegaly	4 (4.3)	1/4 (25)	0.12
Laboratory characteristics			
Platelets count (x10^3^/µL)			
<150	37 (40.2)	3/37 (8.1)	0.91
$\geq$150	55 (59.8)	4/55 (4.3)	
Total bilirubin (mg/dL)			
<10	66	1/66 (1.51)	0.008
$\geq$10	27	6/27 (22.22)	
SGOT/AST (U/L) (n = 91)			
<120	40	1/40	0.13
$\geq$120	51	6/51	
SGPT/ALT (U/L) (n = 84)			
<120	27	1/27 (3.70)	0.31
$\geq$120	57	6/57 (10.52)	
ALP (U/L) (n = 90)			
<240	42	2/42 (4.76)	0.32
$\geq$240	48	5/43 (11.62)	
INR (n = 83)			
<1.3	49	1/49 (2.04)	0.059
$\geq$1.3	34	5/29 (17.24)	

For the 92 pregnant patients, there were 96 liver-related diagnoses. Forty-two patients (45.6%) were diagnosed to be having acute viral hepatitis (AVH). Twenty-seven patients (29.3%) formed part of the spectrum of hypertensive disorder of pregnancy including hypertensive disorder of pregnancy, eclampsia, and HELLP syndrome. One patient (1.1%) was diagnosed to be having AFLP. Nine patients (9.8%) were found to have cholestatic jaundice. Two patients (2.2%) had hyperemesis gravidarum. Three patients (3.3%) were diagnosed to be having septicemic hepatitis. Dengue IgM and plasmodium vivax malaria antigen were found to be positive in two patients (2.2%) each. Four patients (4.3%) were leptospira IgM reactive. These four patients had co-infection with HEV. There was one patient (1.1%) with underlying CLD (idiopathic) who was pregnant and formed the part of our study. No etiology could be established in five (5.4%) patients and they were labeled as idiopathic. In our study, there were seven maternal deaths during the hospital stay among the 92 pregnant women with liver-related disease, giving overall maternal mortality of 7.6%. The mortality rates in various liver diseases in pregnancy were (100%) for AFLP, (50%) for complicated malaria, (25%) for co-infection of AVH with leptospira, (7.9%) for AVH alone, and (3.7%) for the hypertensive disorder of pregnancy (Table 4).

**Table 4:** Maternal mortality and age-wise distribution according to various etiological characteristics of liver disease in pregnancy *Four patients had co-infections with hepatitis E virus and leptospira, one patient out of which expired. One patient had combination of hyperemesis gravidarum and AVH, one patient had combination of pregnancy-induced hypertension and AVH. AVH: acute viral hepatitis

Liver-related diagnosis	0-13 weeks	14-28 weeks	29-40 weeks	Postpartum	Total		Maternal mortality	
					No.	%	No.	%
AVH	0	4	37	1	42	45.6	3	7.1
Hypertensive disorder of pregnancy	0	3	24	0	27	29.3	1	3.7
Acute fatty liver of pregnancy	0	0	1	0	1	1.1	1	100
Cholestatic jaundice	0	1	7	1	9	9.8	0	0.0
Hyperemesis gravidarum	0	1	1	0	2	2.2	0	0.0
Septicemic hepatitis	0	1	1	1	3	3.3	0	0.0
Dengue fever	0	0	1	1	2	2.2	0	0.0
Malaria	0	0	2	0	2	2.2	1	50
AVH + leptospirosis*	0	1	3	0	4	4.3	1	25.0
Chronic liver disease	0	0	1	0	1	1.1	0	0.0
Idiopathic	0	0	5	0	5	5.4	0	0.0
Total	0	11	81	4	96	100.0	7	7.6

## Discussion

Various studies have been done to assess the incidence and clinical profile of liver diseases in pregnancy. While pregnancy-specific or pregnancy-predisposed hepatobiliary diseases are well described, their epidemiology among Asian Indians, especially in north India, is not well documented. The epidemiology of most pregnancy-related liver disorders is either unknown or only patchily recorded. The incidence of liver disease in our study was 3.45% with 92 patients diagnosed with liver-related disease among 2,663 pregnant patients admitted during the study period of 18 months.

In a study by Kingham et al. in Southwest Wales, a total of 142 patients (3.24%) had abnormal liver tests [4]. There were 206 contributing diagnoses, with the great majority being pregnancy-specific, and among the most important were pre-eclampsia, followed by HELLP syndrome, obstetric cholestasis, hyperemesis gravidarum, AFLP, and hepatic infarct. Sepsis, postoperative factors, and placental pathology were not uncommonly responsible but incidental or pre-existing hepatobiliary disease was infrequent. A study by Panther et al. concluded that most liver dysfunctions in pregnancy are caused by one of five pregnancy-related liver diseases, aside from pre-existing liver diseases that develop before pregnancy, such as gall stones or viral hepatitis [5]. Complications including acute renal failure and disseminated intravascular coagulation are also associated with HELLP syndrome. This increases morbidity and mortality, both in the mother and fetus. It has been proposed that AFLP and HELLP are parts of the same spectrum, and both lead to multi-organ involvement and serious complications [6]. AFLP was seen in one of our patients who had encephalopathy, renal failure, and coagulopathy, and it was associated with both maternal and perinatal mortality. Western studies have shown maternal mortality of up to 13% and perinatal mortality of up to 9% in patients with AFLP, while in Indian reports, these have been 31-54% and 100%, respectively [4,7-9].

Hepatitis E is known to be the most common cause of AVH during pregnancy in India (58-86%) [10-12]. In our study, HEV was the most common cause (97.6%) of acute hepatitis and was seen predominantly in the third trimester. HEV in pregnancy is often complicated by fulminant hepatic failure and high maternal and perinatal mortality [11-13]. In patients with cirrhosis, pregnancy is stated to be rare because of the resulting anovulation. We encountered one pregnant woman with CLD. No ascites, variceal bleed, or encephalopathy was present. Disorders unrelated to pregnancy were not uncommon and could be differentiated by appropriate tests. Two patients had falciparum malaria with multi-organ failure, and one patient had maternal death. There were two patients with dengue fever, and it was associated with 100% perinatal mortality. Four patients had combined AVH and leptospirosis and it was associated with one maternal and two perinatal deaths.

Maternal mortality in pregnancy with liver disease has dropped to 1.1% in recent studies from western countries [1,4]. The study by Rathi et al. observed that overall maternal and perinatal mortality rates were 19.7% and 35.4%, respectively [9]. The prospective study by Ch’ng et al. observed that there were no maternal deaths due to liver dysfunction in the first or second trimester. Liver dysfunction in the third trimester was associated with serious consequences. The overall and perinatal mortality was 20.2% and 24.6%, respectively [4]. Furthermore, they observed that despite substantial liver-related morbidity, there were no maternal deaths, and only two intrauterine deaths occurred [4]. A study by Tank et al. identified the severe liver disease in 0.42% of deliveries. Maternal and perinatal mortality were 42.3% and 61.5%, respectively [14].

In our study population, the overall maternal and perinatal mortality were 7.6% and 25.0%, respectively. These differences may reflect differences in referral patterns in various institutions apart from genetic and socioeconomic differences in cohorts.

## Conclusions

To conclude, our study shows that liver disease is relatively common in Indian pregnant women. It is associated with high maternal and perinatal mortality, even in a tertiary referral center. A high index of suspicion of liver disease, early diagnosis, prompt referral to a higher center when required, appropriate supportive management, and a proactive policy of early delivery when indicated may improve the maternal and perinatal outcomes in pregnant women with liver disease.
